# Au nanostructure arrays for plasmonic applications: annealed island films versus nanoimprint lithography

**DOI:** 10.1186/s11671-015-0819-1

**Published:** 2015-03-01

**Authors:** Andrii M Lopatynskyi, Vitalii K Lytvyn, Volodymyr I Nazarenko, L Jay Guo, Brandon D Lucas, Volodymyr I Chegel

**Affiliations:** Department of Functional Optoelectronics, V. E. Lashkaryov Institute of Semiconductor Physics NASU, 41 Nauki avenue, 03028 Kyiv, Ukraine; Department of Molecular Immunology, Laboratory of Nanobiotechnology, O. V. Palladin Institute of Biochemistry NASU, 9 Leontovycha street, 01601 Kyiv, Ukraine; Department of Electrical Engineering and Computer Science, University of Michigan, 1301 Beal Avenue, MI 48109-2122 Ann Arbor, USA

**Keywords:** Localized surface plasmon resonance, Nanostructures, Annealed island films, Nanoimprint lithography, Plasmonic enhancement, 81.07.-b, 73.20.Mf

## Abstract

This paper attempts to compare the main features of random and highly ordered gold nanostructure arrays (NSA) prepared by thermally annealed island film and nanoimprint lithography (NIL) techniques, respectively. Each substrate possesses different morphology in terms of plasmonic enhancement. Both methods allow such important features as spectral tuning of plasmon resonance position depending on size and shape of nanostructures; however, the time and cost is quite different. The respective comparison was performed experimentally and theoretically for a number of samples with different geometrical parameters. Spectral characteristics of fabricated NSA exhibited an expressed plasmon peak in the range from 576 to 809 nm for thermally annealed samples and from 606 to 783 nm for samples prepared by NIL. Modelling of the optical response for nanostructures with typical shapes associated with these techniques (parallelepiped for NIL and semi-ellipsoid for annealed island films) was performed using finite-difference time-domain calculations. Mathematical simulations have indicated the dependence of electric field enhancement on the shape and size of the nanoparticles. As an important point, the distribution of electric field at so-called ‘hot spots’ was considered. Parallelepiped-shaped nanoparticles were shown to yield maximal enhancement values by an order of magnitude greater than their semi-ellipsoid-shaped counterparts; however, both nanoparticle shapes have demonstrated comparable effective electrical field enhancement values. Optimized Au nanostructures with equivalent diameters ranging from 85 to 143 nm and height equal to 35 nm were obtained for both techniques, resulting in the largest electrical field enhancement. The application of island film thermal annealing method for nanochips fabrication can be considered as a possible cost-effective platform for various surface-enhanced spectroscopies; while the NIL-fabricated NSA looks like more effective for sensing of small-size objects.

## Background

Plasmonic phenomena are widely used in optical devices [1], imaging microscopy [2], biosensing [3-5], and medical diagnostics [6-8]. Improvement of sensitivity, even down to single molecule detection limits, is needed in many applications, and this problem demands a solution at the present moment. One of the possible ways to obtain general sensitivity enhancement for multiple applications is to fabricate nanopatterned plasmonic substrates capable of generating strong local electromagnetic fields, or, in other words, offering significant plasmonic enhancement (PE), due to occurrence of localized surface plasmon resonance (LSPR) phenomenon in highly conductive metal nanoparticles. It was shown both theoretically and experimentally that enhanced local field provides signal amplification for LSPR [9-11], surface-enhanced Raman scattering (SERS) [10,12], surface-enhanced fluorescence (SEF) [13-15], and surface-enhanced infrared absorption (SEIRA) [16,17] techniques. The peculiarity of PE accompanying LSPR is that enhanced field is concentrated in confined space with nanometer dimensions (‘hot spots’) [18] phenomenon, which depends on nanostructure size, shape, and material properties [19,20].

Nanostructures enabling the PE effect can be fabricated using a multitude of methods [21]. A simple and commonly used approach involves highly conductive, continuous film possessing surface roughness as effective plasmonic amplifiers; however, this method does not yield the ability for spectral tuning, and, consequently, the process of enhancement cannot be applied for matching with molecular resonances that is preferable for a number of spectroscopic techniques. The benefit of spectrally controlled nanostructured PE surfaces is obvious as only uniform surface-bound nanostructure arrays (nanochips) with known surface 3D geometry can provide a real possibility to perform preliminary estimation of final PE parameters when using this technique and ensure their stability and reproducibility.

In this present work, two different approaches for nanostructure fabrication were used - a method based on gold island film deposition with subsequent thermal annealing and nanoimprint lithography (NIL) technique. The most evident advantage of the latter method is an exploitation of templates with relatively large linear dimensions and sub-10-nm resolution [22] for nanostructure preparation that makes NIL suitable for fabrication of uniformly oriented and homogeneous gold nanostructure arrays (NSA) with controlled nanoparticle size, shape, and spacing. However, due to the associated high costs for NIL technique exploitation in terms of equipment needed for NIL template fabrication and the nanoimprinting process itself, the development of an alternative technique which can be the basis for PE nanochip is of considerable interest. Here, we compare the two abovementioned methods of Au NSA fabrication from the point of view of LSPR spectral measurements and PE modelling.

## Methods

### Fabrication of random NSA samples

**Figure 1:**
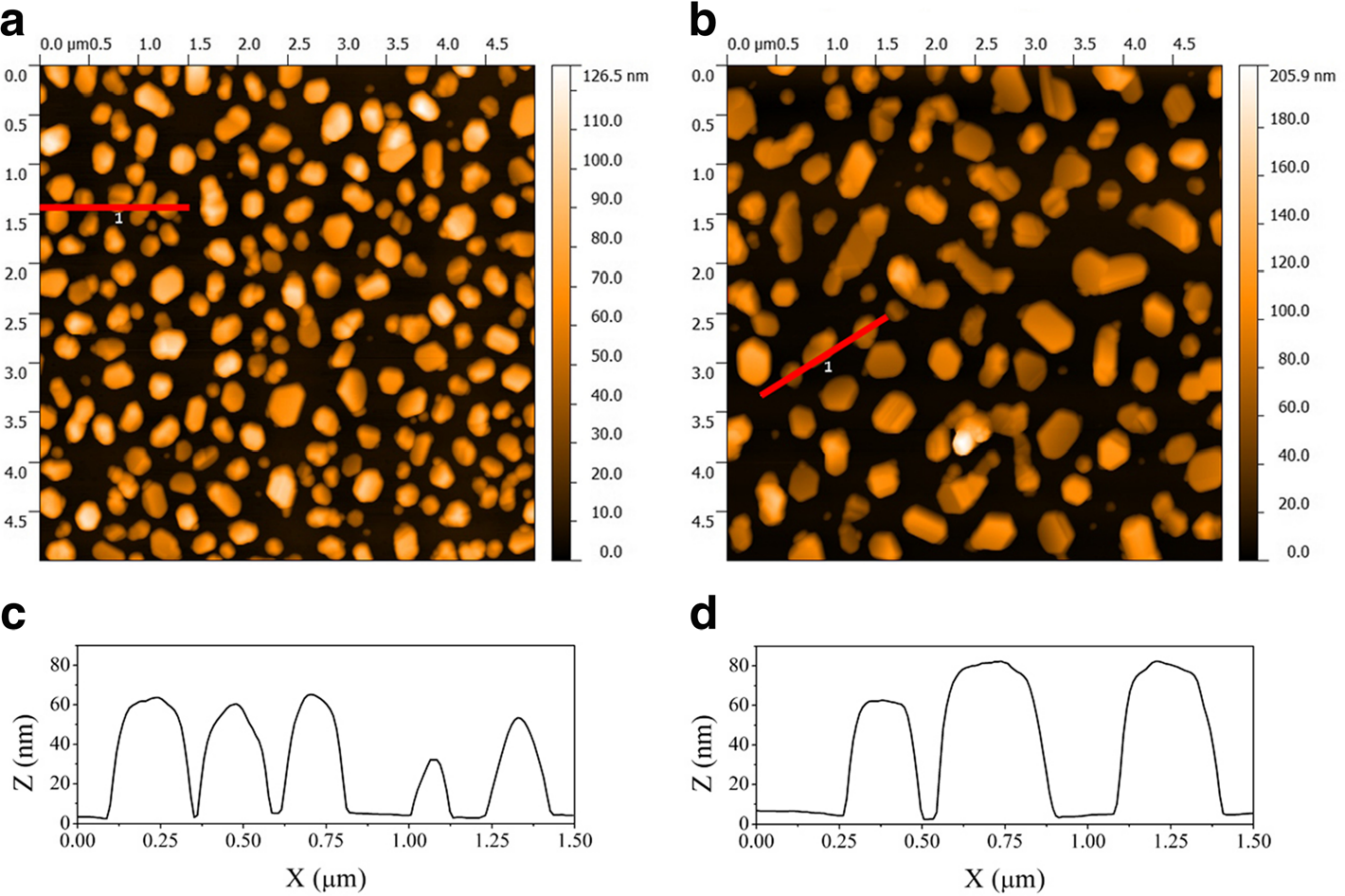
**AFM characterization results for random gold NSA samples produced via thermal annealing of gold island films.** **(a,** **b)** Top view AFM images and **(c,** **d)** corresponding AFM profiles for random gold NSA samples R6 and R7, respectively (see Table 1).

We have adapted the PE nanochip fabrication technique based on gold island film deposition with subsequent thermal annealing [23], which is an affordable NSA preparation method yielding satisfactory results. Briefly, gold island films of varying thickness (5 to 15 nm) were obtained by thermal evaporation in vacuum on precleaned microscope glass substrates (*n* = 1.51). After island film deposition, samples were annealed at 550°С for 6 h in N_2_ atmosphere. As a result of annealing, the gold film color changed from blue of different intensity to blue, violet, and pink (depending on the film thickness) that confirms the formation of separated gold nanoparticles having different sizes depending on the initial island film thickness values (see atomic force microscopy (AFM) images in Figure 1).

### Fabrication of ordered NSA samples

Ordered gold nanoparticle arrays were produced by means of NIL technique according to the protocol described earlier [24,25]. Briefly, Pyrex glass substrates were cleaned in a 1:1 piranha solution (30% H_2_O_2_:29% NH_4_OH), rinsed with a copious amount of DI:H_2_O, and dried using N_2_. The resist was spincoated to the appropriate thickness on the substrates, baked for solvent removal, and imprinted using the template. After nanoimprinting and sample separation, the residual polymer layer was removed using O_2_ plasma reactive-ion etching. Au metallization was accomplished using an electron beam evaporator. Lift-off was performed by soaking the samples in acetone and using an ultrasonic bath. After completion of lift-off, samples were rinsed with methanol and isopropanol and dried with N_2_. This technique can be used to create a variety of structures by simply using different one-dimensional gratings (i.e., with various duty cycles or periods) and relative angular orientation of the gratings for successive imprints to create different NIL templates [24].

**Figure 2:**
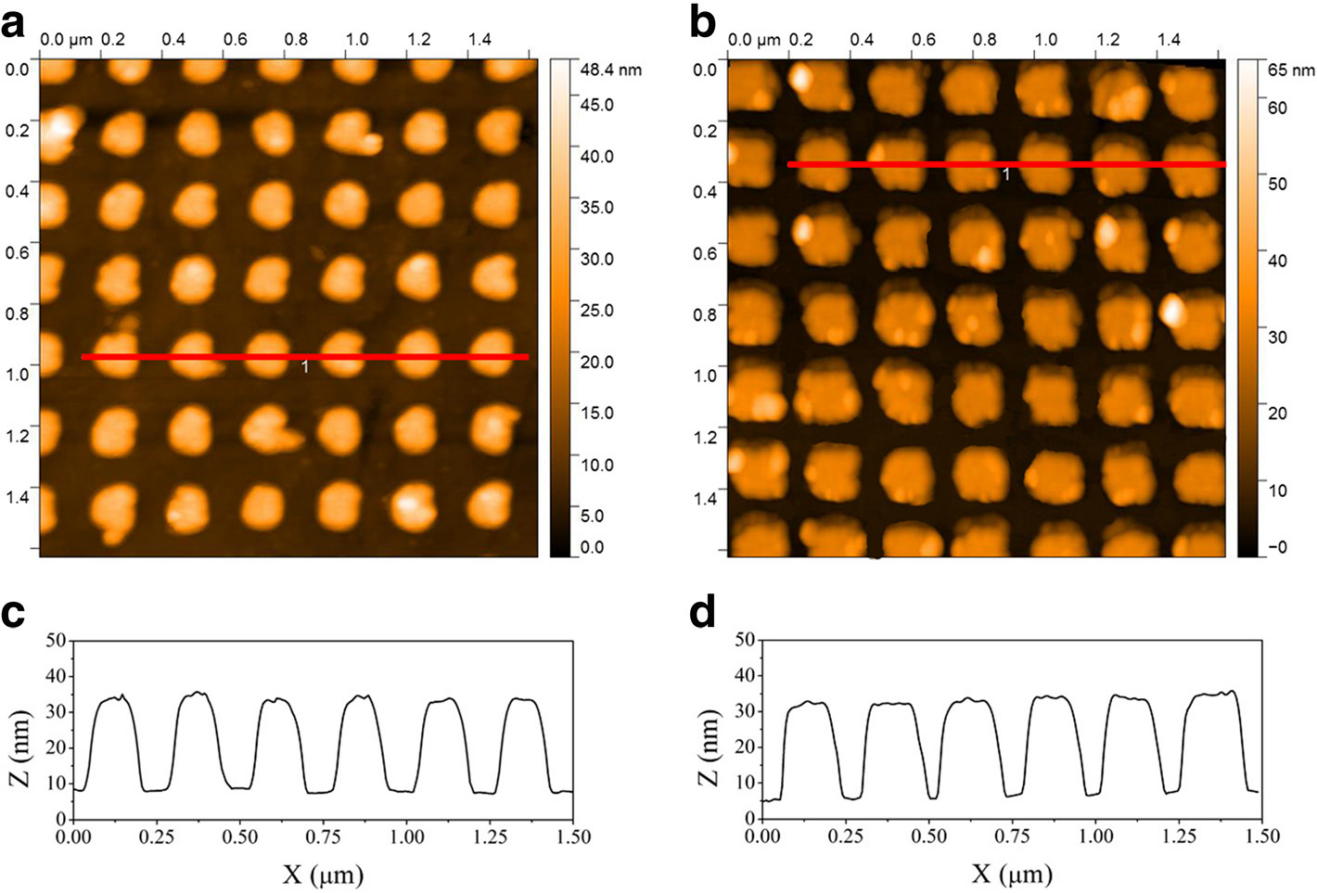
**AFM characterization results for ordered gold NSA samples produced using NIL.** **(a,** **b)** Top view AFM images and **(c,** **d)** corresponding AFM profiles for ordered gold NSA samples O2 and O4, respectively (see Table 2).

Structural characteristics of ordered NSA were investigated using atomic force microscopy method. NIL-fabricated NSA samples of different geometry were studied. These samples were comprised of parallelepiped-shaped nanoparticles, located in an ordered array with square or rectangular lattice. AFM images of nanostructure arrays fabricated using the mentioned technique are shown in Figure 2.

### AFM measurements

Atomic force microscope ‘NanoScope IIIa Dimension 3000’ (Digital Instruments/Bruker, Santa Barbara, CA, USA) was used to study the NSA sample morphology. AFM data treatment in order to obtain NSA structural characteristics was performed using Gwyddion 2.37 software.

### Spectrophotometric measurements

Light extinction measurements were carried out using a compact localized surface plasmon resonance spectrometer ‘NanoPLASMON-003’ (NanoPlasmon Devices, Elmhurst, IL, USA) possessing a spectral range of 400 to 1,100 nm and compatible with nanochips of various size (within the limits of a 1 × 3 inch standard plain microscopic slide) in both stationary and flow real-time operation modes. Unpolarized light from a tungsten-halogen light source was incident normally to the nanochip surface and collected using an optical fiber connected to a built-in miniature spectrometer.

### Light extinction and field intensity enhancement theoretical modelling

To optimize the parameters required to achieve the highest PE response for the random and ordered NSAs, a theoretical study of intensity enhancement for electric component of electromagnetic field near gold nanostructure surface depending on its shape and size was carried out. According to AFM results (see Figure 1), nanoparticles of random NSA can be considered approximately as two-axial semi-ellipsoid in the case of smaller nanoparticles and more complex shapes in the case of larger ones. Certainly, we are aware that to fully simulate the random NSA plasmonic properties, a complete reproduction of nanostructure shape and size for each of the nanoparticle arrays is needed, which would also provide a possibility to correctly include interparticle electromagnetic interactions into the model. However, the implementation of such an approach requires costly computational resources and lengthy simulation times. To work around these challenges, we apply a common nanoparticle model for random NSA in the shape of two-axial semi-ellipsoid with average dimensions presented in Table 1.

**Table 1:** **Spectral and structural characteristics of random NSA samples produced via thermal annealing of gold island films**

**NSA sample**	**Peak position in extinction spectrum (nm)**	**Average equivalent diameter (nm)**	**Average height (nm)**	**Average interparticle distance (nm)**
R1	576	56	27	93
R2	582	56	28	104
R3	585	85	35	137
R4	604	143	35	216
R5	633	152	47	228
R6	680	235	53	346
R7	733	330	61	414
R8	776	470	91	576
R9	809	524	114	647

Sample numbers follow the initial gold island film thickness increase.

It is well known that the greatest electric field intensity enhancements associated with highly conductive metal nanoparticles are expected in ‘hot spots’ on the nanoparticle surface, which are usually located on sharp corners, edges, or projections of nanoparticles [18-20]. As the semi-ellipsoid shape has no expressed regions for ‘hot spots,’ we anticipate the NIL method allows for the production of NSAs with the abovementioned features resulting from nanoparticles of various shapes (including parallelepiped-shaped). To compare the ‘hot spots’ influence on the PE level, the nanoparticles having the parallelepiped shape with a square base of the same area (i.e., having the same equivalent diameter) and same height (effective geometrical parameters (EGP)) as their semi-ellipsoid-shaped counterparts were included in the simulation. Due to the relatively sparse character of investigated NSA samples (surface-to-surface distance between nanostructures lies within the 37- to 123-nm range as determined by AFM), the nanoparticles in the array were considered to be non-interacting (the strongest coupling between nanoparticles was observed at surface-to-surface distance close to 20 nm [26]) and the individual Au nanoparticles have been selected for theoretical calculations. We are aware that the modelling of single nanostructures does not take into account the possible coupling between the adjacent nanoparticles. On the other hand, some researches require the characterization of single nanoparticles [27,28]. However, modelling nanoparticle arrays, where cooperative plasmon modes involving multiple nanoparticles exist (e.g., dipole-dipole or higher multipole interactions), is under way, and its results will be presented in a future work.

Finally, the Au nanoparticles of semi-ellipsoid and parallelepiped shape located on glass substrates in air environment have been considered as our model system. Due to the lack of a general analytical model that fully describes the electromagnetic response of arbitrary-shaped nanoparticle, numeric methods should be used. Thus, to estimate the scattering and extinction of electromagnetic field by non-spherical Au nanoparticles, finite-difference time-domain (FDTD) method implemented in Lumerical’s FDTD Solutions software was exploited. This allowed spectral distributions of local electromagnetic field intensity in the near-field of the nanoparticle and light extinction spectrum that represent the excitation of LSPR in the nanoparticle to be obtained. The full 3D FDTD model was computed for linearly polarized incident light and wavelength ranging from 400 to 2,000 nm with simulation mesh fixed at 2 × 2 × 2 nm. Linearly polarized light source was used, which is common for the case of symmetric nanoparticle shapes considered; therefore, results obtained can also be applied to unpolarized NSA illumination.

## Results and discussion

### Characteristics of random NSA samples

**Figure 3:**
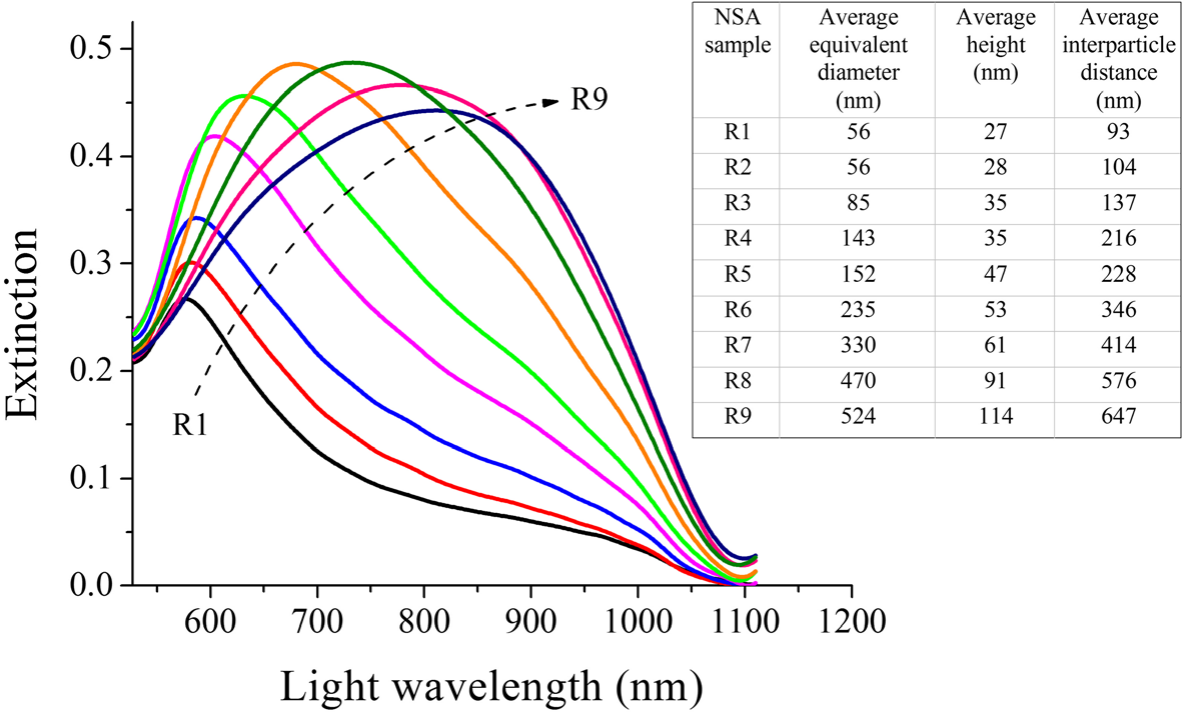
**Measured extinction spectra of random NSA samples produced via thermal annealing of gold island films.** Normal incidence unpolarized light extinction spectra for samples R1 to R9 with different initial gold island film thickness exhibit peaks located in the wavelength range from 576 to 809 nm. Inset: the table with respective NSA geometrical parameters.

As a result of spectrophotometric measurements, light extinction spectra of the samples in air atmosphere were obtained (Figure 3). It was found that the peak position in the unpolarized light extinction spectrum, which corresponds to the occurrence of LSPR, shifts towards longer wavelengths with an increase in the initial gold island film thickness (see Figure 3 and Table 1). Additionally, the direct relation between lateral dimensions and height of nanostructures produced after annealing and the initial gold island film thickness was revealed. Thus, it is possible to tune the LSPR spectral position in the wavelength range from 576 to 809 nm (see Figure 3), and the associated PE spectral profile, by changing the initial gold island film thickness. Obviously, considered PE nanochips fabrication technology based on gold island films with subsequent thermal annealing can be exploited while taking into account inherent technological limitations that hinder the preparation of geometrically ordered nanoparticle arrays.

### Characteristics of ordered NSA samples

**Figure 4:**
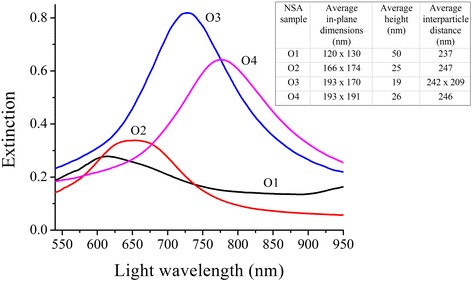
**Measured extinction spectra of ordered NSA samples produced using NIL.** Normal incidence unpolarized light extinction spectra for samples O1 to O4 exhibit peaks located in the wavelength range from 606 to 783 nm. Inset: the table with respective NSA geometrical parameters.

Spectral characteristics and structural parameters of ordered NSAs were obtained using the previous experimental conditions and yielded the following results (Figure 4, Table 2). Unpolarized light extinction spectra for each of the samples exhibited bands having an expressed peak with different spectral positions (from 606 to 783 nm) and extinction intensities, which mainly depend on the geometrical parameters of the nanostructures. Significant spectral shift of the LSPR band also implies the possibility of wide-range wavelength tuning using NSA fabricated by the NIL technique. Strong variation of extinction intensity should be noted as a disadvantage of such spectral tuning for both methods of NSA fabrication.

**Table 2:** **Spectral and structural characteristics of ordered NSA samples produced using NIL**

**NSA sample**	**Peak position in extinction spectrum (nm)**	**Average in-plane dimensions (nm)**	**Average height (nm)**	**Average interparticle distance (nm)**
O1	606	120 × 130	50	237
O2	652	166 × 174	25	247
O3	731	193 × 170	19	242 × 209
O4	783	193 × 191	26	246

### Modelling results

**Figure 5:**
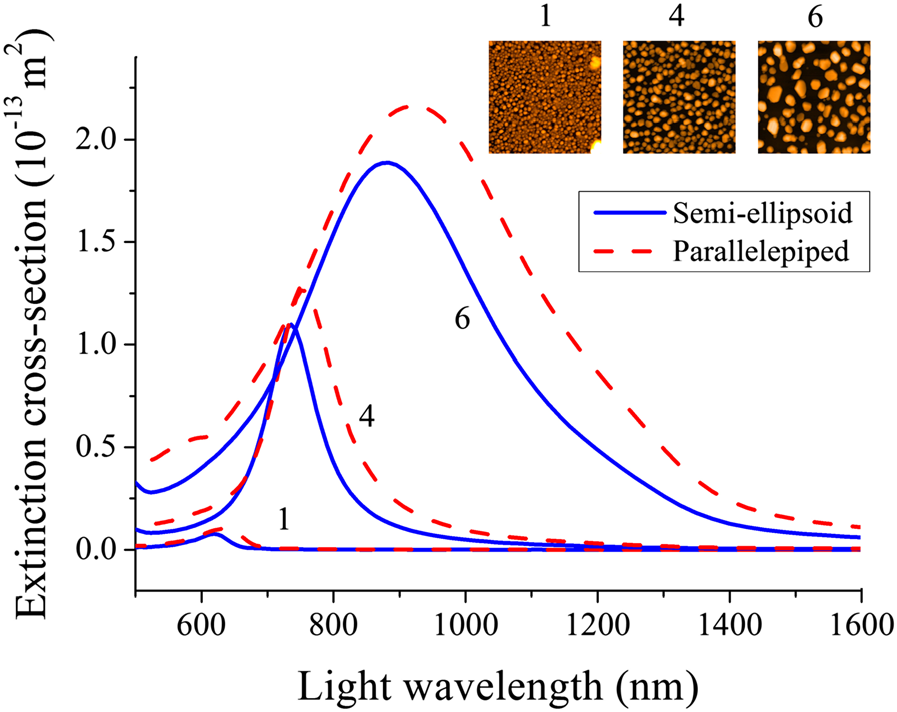
**Simulated light extinction cross-section spectra for semi-ellipsoid- and parallelepiped-shaped Au nanoparticles on glass substrates.** Insets show top view AFM images for random gold NSA samples. Numbers correspond to samples R1, R4, and R6, respectively. Effective geometrical parameters of Au nanoparticles used in FDTD modelling are the same for semi-ellipsoid and parallelepiped shapes and correspond to the data presented in Table 1 for samples R1, R4, and R6, respectively.

First, light extinction properties of model nanoparticle systems have been simulated for the two considered shapes (i.e., semi-ellipsoid and parallelepiped). Figure 5 shows typical light extinction spectra for both shapes with EGP corresponding to samples R1, R4, and R6 presented in Table 1. It is evident that simulated extinction spectra follow the same trend of red-shifting and widening upon the increase in nanoparticle dimensions as experimental extinction spectra, which can be related to the mutual influence of light scattering and secondary radiation of electrons (radiation damping effect) [29,30]. Additionally, extinction response of semi-ellipsoid and parallelepiped-shaped nanoparticles does not exhibit noticeable differences except for a permanent red-shift and marginally increased FWHM of parallelepiped-shaped nanoparticle spectrum (see Figure 5), which can be attributed to the shape effect. Observed difference between the experimental and modelled extinction spectra, which is most evident for random NSA, can be explained by the growing mismatch between the model semi-ellipsoid and experimental nanostructure shapes with increasing nanostructures size. Additionally, the near-field interaction between nanostructures and their size distribution also contribute to the observed discrepancy between the experimental and calculated data. Nevertheless, the abovementioned trend of spectra red-shifting and widening is obvious.

**Figure 6:**
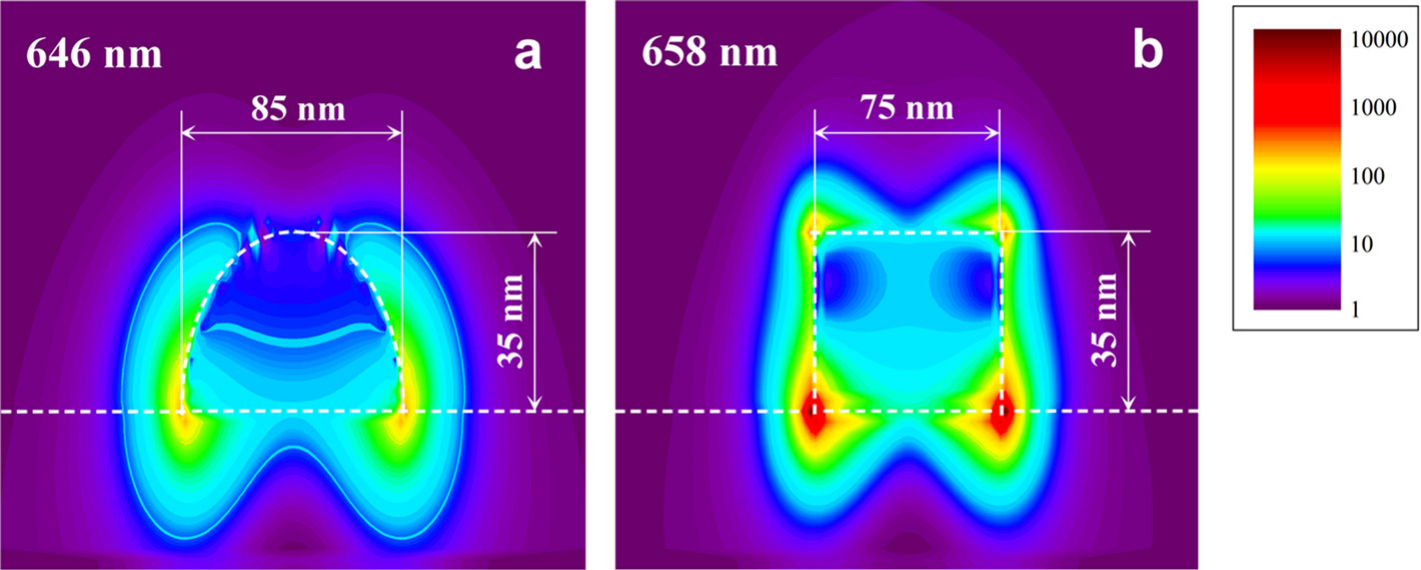
**Simulated profiles of electric field intensity enhancement for differently shaped Au nanoparticles.** Effective geometrical parameters of **(a)** semi-ellipsoid- and **(b)** parallelepiped-shaped Au nanoparticles on glass substrates correspond to sample R3 (see Table 1). Electric field profiles correspond to light wavelengths of 646 and 658 nm for semi-ellipsoid- and parallelepiped-shaped Au nanoparticles, respectively, which provide the maximal intensity enhancement. Electric field polarization and wave vectors lie in the profile plane under the normal incidence conditions. Dashed lines indicate nanoparticle and substrate borders. Different nanoparticle widths are shown due to the exploited model with identical nanoparticle base area for semi-ellipsoid- and parallelepiped-shaped Au nanoparticles (see ‘Methods’ section). Vertical dimension scale is increased for better visual perception.

Comparison of maximal local electric field distribution around the nanoparticles of different shapes shows that parallelepiped-shaped nanoparticles on glass substrates provide electric field intensity enhancements more than an order of magnitude greater than their semi-ellipsoidal counterparts (Figure 6). For example, parallelepiped nanoparticles with equivalent diameters of 85 nm and height of 35 nm exhibit maximal incident field intensity enhancement up to approximately 1.2 × 10^4^. In comparison, semi-ellipsoidal nanoparticles with the same EGP provide maximal electric field intensity enhancement of only about 4.1 × 10^2^.

**Figure 7:**
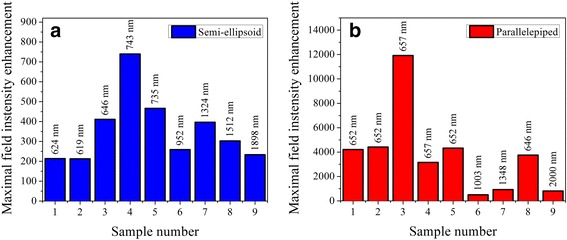
**Calculated values of maximal electric field intensity enhancement near differently shaped Au nanoparticles.** Simulation results correspond to **(a)** semi-ellipsoid- and **(b)** parallelepiped-shaped Au nanoparticles on glass substrates. Effective geometrical parameters of Au nanoparticles used in FDTD modelling are the same for semi-ellipsoid and parallelepiped shapes and correspond to the data presented in Table 1 for samples R1 to R9, respectively. Labels above the bars indicate the light wavelength, which the maximal electric field intensity enhancement for specific nanoparticle geometry was obtained at.

**Figure 8:**
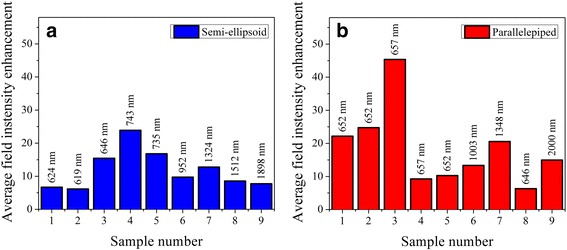
**Calculated values of average electric field intensity enhancement within a 25**-**nm vicinity of differently shaped Au nanoparticles.** Simulation results correspond to **(a)** semi-ellipsoid- and **(b)** parallelepiped-shaped Au nanoparticles on glass substrates. Effective geometrical parameters of Au nanoparticles used in FDTD modelling are the same for semi-ellipsoid and parallelepiped shapes and correspond to the data presented in Table 1 for samples R1 to R9, respectively. Labels above the bars indicate the light wavelength, which the maximal electric field intensity enhancement for specific nanoparticle geometry was obtained at.

The whole set of maximal electric field enhancement values obtained for each of the nanoparticle geometries is presented in Figure 7. It is evident that the largest enhancement values are exhibited when equivalent diameters of both types of nanoparticles range from 85 to 143 nm and the height of nanoparticles is 35 nm. These optimized geometrical parameters can be used for manufacturing purposes to fabricate Au NSA providing plasmonic enhancement for different applications. For a defined nanoparticle shape and geometrical parameters, maximal electric field intensity enhancement is obtained at a specific light wavelength, which was found to range from about 620 nm (for small nanoparticles) to near infrared region (for large nanoparticles). Detailed light wavelength values are contained in labels in Figures 6, 7, and 8.

It is particularly important to discuss the difference between electric field intensity enhancements for semi-ellipsoidal and parallelepiped nanoparticles considering not only the ‘hot spots’ regions but the vicinity of the entire nanoparticle surface where other objects of interest, e.g., molecular or biomolecular species, can reside. To accomplish this task, we calculated the average electric field intensity enhancements within a 25-nm vicinity (maximal value for a biomolecular monolayer) of both semi-ellipsoidal and parallelepiped Au nanoparticles (Figure 8). Interestingly, in this case, both nanoparticle shapes have demonstrated comparable average electric field enhancement values that peaked at about 24 and 45 for semi-ellipsoidal and parallelepiped nanoparticles, respectively. This result implies that not only NIL-fabricated nanochips but also NSA samples obtained using island film thermal annealing method can be promising as a possible PE basis for various surface-enhanced spectroscopies. The NIL NSA can be more effective for detection of small size molecules, which are able to reach ‘hot spots’ in the vicinity of nanostructure base.

## Conclusions

Samples of random and ordered gold nanoparticle arrays with different morphologies, which were fabricated using thermal annealing of vacuum-evaporated island films and nanoimprint lithography methods, exhibit differences in the maximal level of plasmonic enhancement. However, both nanoparticle shapes have shown comparable average enhancement values in their near vicinity. Among the considered configurations, optimal enhancement is achieved when Au nanoparticles have equivalent diameters ranging from 85 to 143 nm and height equal to 35 nm. Both techniques allow the wavelength tuning of LSPR for considered NSA samples in the range of about 200 nm. Thermally annealed island films revealed smooth nanostructure shapes resembling semi-ellipsoids, with easy access to their surfaces by detected molecules possessing a wide range of sizes. In contrast, nanoimprint lithography produced nanostructures with sharp corners that can, on the one hand, generate ‘hot spots’ with strong PE, and, on the other hand, hinder the access of larger analyte molecules to these regions thereby creating more favorable conditions for small molecule detection. In summary, both studied fabrication approaches can find different implementations to prepare the nanostructured plasmonic arrays for a wide range of applications, such as biosensing and resonant methods using electromagnetic fields of nanostructures.

## Abbreviations

AFM: atomic force microscopy
EGP: effective geometrical parameters
FDTD: finite-difference time-domain method
LSPR: localized surface plasmon resonance
NIL: nanoimprint lithography
NSA: nanostructure array
PE: plasmonic enhancement
SEF: surface-enhanced fluorescence
SEIRA: surface-enhanced infrared absorption
SERS: surface-enhanced Raman scattering

## References

[1] Kildishev AV, Boltasseva A, Shalaev VM (2013). Planar photonics with metasurfaces. Science.

[2] Gjonaj B, Aulbach J, Johnson PM, Mosk AP, Kuipers L, Lagendijk A (2013). Focusing and scanning microscopy with propagating surface plasmons. Phys Rev Lett.

[3] Bauch M, Toma K, Toma M, Zhang Q, Dostalek J (2014). Plasmon-enhanced fluorescence biosensors: a review. Plasmonics.

[4] Ruemmele JA, Hall WP, Ruvuna LK, Van Duyne RP (2013). A localized surface plasmon resonance imaging instrument for multiplexed biosensing. Anal Chem.

[5] Anker JN, Hall WP, Lambert MP, Velasco PT, Mrksich M, Klein WL (2009). Detection and identification of bioanalytes with high resolution LSPR spectroscopy and MALDI mass spectrometry. J Phys Chem C.

[6] Sciacca B, François A, Klingler-Hoffmann M, Brazzatti J, Penno M, Hoffmann P (2013). Radiative-surface plasmon resonance for the detection of apolipoprotein E in medical diagnostics applications. Nanomedicine.

[7] Vo-Dinh T, Fales AM, Griffin GD, Khoury CG, Liu Y, Ngo H (2013). Plasmonic nanoprobes: from chemical sensing to medical diagnostics and therapy. Nanoscale.

[8] Haes AJ, Hall WP, Chang L, Klein WL, Van Duyne RP (2004). A localized surface plasmon resonance biosensor: first steps toward an assay for Alzheimer’s disease. Nano Lett.

[9] Dantham VR, Holler S, Barbre C, Keng D, Kolchenko V, Arnold S (2013). Label-free detection of single protein using a nanoplasmonic-photonic hybrid microcavity. Nano Lett.

[10] Henry AI, Bingham JM, Ringe E, Marks LD, Schatz GC, Van Duyne RP (2011). Correlated structure and optical property studies of plasmonic nanoparticles. J Phys Chem C.

[11] Haes AJ, Zou S, Schatz GC, Van Duyne RP (2004). A nanoscale optical biosensor: the long range distance dependence of the localized surface plasmon resonance of noble metal nanoparticles. J Phys Chem B.

[12] Genov DA, Sarychev AK, Shalaev VM, Wei A (2004). Resonant field enhancements from metal nanoparticle arrays. Nano Lett.

[13] Deng W, Xie F, Baltarac HTMCM, Goldys EM (2013). Metal-enhanced fluorescence in the life sciences: here, now and beyond. Phys Chem Chem Phys.

[14] Zhang Z, Yang P, Xu H, Zheng H (2013). Surface enhanced fluorescence and Raman scattering by gold nanoparticle dimers and trimers. J Appl Phys.

[15] Xu S, Cao Y, Zhou J, Wang X, Wang X, Xu W (2011). Plasmonic enhancement of fluorescence on silver nanoparticle films. Nanotechnology.

[16] Brown LV, Zhao K, King N, Sobhani H, Nordlander P, Halas NJ (2013). Surface-enhanced infrared absorption using individual cross antennas tailored to chemical moieties. J Am Chem Soc.

[17] Dovbeshko GI, Chegel VI, Gridina NY, Repnytska OP, Shirshov YM, Tryndiak VP (2002). Surface enhanced IR absorption of nucleic acids from tumor cells: FTIR reflectance study. Biopolymers.

[18] Sánchez-Gil JA, García-Ramos JV, Méndez ER (2000). Near-field electromagnetic wave scattering from random self-affine fractal metal surfaces: Spectral dependence of local field enhancements and their statistics in connection with surface-enhanced Raman scattering. Phys Rev B.

[19] Weeber JC, Girard C, Krenn JR, Dereux A, Goudonnet JP (1999). Near-field optical properties of localized plasmons around lithographically designed nanostructures. J Appl Phys.

[20] Hao E, Schatz GC (2004). Electromagnetic fields around silver nanoparticles and dimers. J Chem Phys.

[21] Chegel VI. Nanostructured materials for biosensor applications: comparative review of preparation methods. In: Ariga K, editor. O’Brien P, Kroto H, Nuzzo R, series editors. Manipulation of nanoscale materials: an introduction to nanoarchitectonics. RSC Nanoscience & Nanotechnology, vol. 24. Cambridge: The Royal Society of Chemistry; 2012. p. 318-55

[22] Chou SY, Krauss PR (1997). Imprint lithography with sub-10 nm feature size and high throughput. Microelectron Eng.

[23] Kedem O, Tesler AB, Vaskevich A, Rubinstein I (2011). Sensitivity and optimization of localized surface plasmon resonance transducers. ACS Nano.

[24] Lucas BD, Kim JS, Chin C, Guo LJ (2008). Nanoimprint lithography based approach for the fabrication of large-area, uniformly-oriented plasmonic arrays. Adv Mater.

[25] Chegel V, Lucas B, Guo J, Lopatynskyi A, Lopatynska O, Poperenko L (2009). Detection of biomolecules using optoelectronic biosensor based on localized surface plasmon resonance. Nanoimprint lithography approach. Semicond Phys Quantum Electron Optoelectron.

[26] Kinnan MK, Chumanov G (2010). Plasmon coupling in two-dimensional arrays of silver nanoparticles: II. Effect of the particle size and interparticle distance. J Phys Chem C.

[27] Chen Y, Munechika K, Ginger DS (2007). Dependence of fluorescence intensity on the spectral overlap between fluorophores and plasmon resonant single silver nanoparticles. Nano Lett.

[28] Rycenga M, Xia X, Moran CH, Zhou F, Qin D, Li ZY (2011). Generation of hot spots with silver nanocubes for single-molecule detection by surface-enhanced Raman scattering. Angew Chem Int Ed Engl.

[29] Noguez C (2005). Optical properties of isolated and supported metal nanoparticles. Opt Mater.

[30] Hu M, Novo C, Funston A, Wang H, Staleva H, Zou S (2008). Dark-field microscopy studies of single metal nanoparticles: understanding the factors that influence the linewidth of the localized surface plasmon resonance. J Mater Chem.

